# Reward Modulates Unconsciously Triggered Adaptive Control Processes

**DOI:** 10.1177/20416695211073819

**Published:** 2022-02-14

**Authors:** Liuting Diao, Wenping Li, Wenhao Chang, Qingguo Ma

**Affiliations:** Business School, 47862 Ningbo University, Ningbo, China; Prudence College, 177546 Zhejiang Business Technology Institute, Ningbo, China; Continuing Education College, 47862 Ningbo University, Ningbo, China; School of Management, 12377 Zhejiang University, Hangzhou, China

**Keywords:** reward, adaptive control processes, consciousness, masked priming task

## Abstract

Adaptive control (e.g., conflict adaptation) refers to dynamic adjustments of cognitive control processes in goal-directed behavior, which can be influenced by incentive rewards. Recently, accumulating evidence has shown that adaptive control processes can operate in the absence of conscious awareness, raising the question as to whether reward can affect unconsciously triggered adaptive control processes. Two experiments were conducted to address the question. In Experiment 1, participants performed a masked flanker-like priming task manipulated with high- and low-value performance-contingent rewards presented at the block level. In this experiment conflict awareness was manipulated by masking the conflict-inducing stimulus, and high- or low-value rewards were presented at the beginning of each block, and participants earned the reward contingent upon their responses in each trial. We observed a great conflict adaptation for high-value rewards in both conscious and unconscious conflict tasks, indicating reward-induced enhancements of consciously and unconsciously triggered adaptive control processes. Crucially, this effect still existed when controlling the stimulus-response repetitions in a rewarded masked Stroop-like priming task in Experiment 2. The results endorse the proposition that reward modulates unconsciously triggered adaptive control to conflict, suggesting that individuals may enable rewarding stimuli to dynamically regulate concurrent control processes based on previous conflict experience, regardless of whether the previous conflict was experienced consciously.

## Introduction

There is general agreement that reward and cognitive control jointly determine human behavior. Reward is generally considered to be an effective motivator of human performance (Botvinick & Braver, 2015; Yee & Braver, 2018), while cognitive control refers to a set of high-order processes that are deemed to flexibly direct behavior in accordance with internal goals and the current context, which allows people to ignore irrelevant stimulus information while focusing attention on goal-relevant parts (Botvinick et al., 2001; Egner, 2008). Moreover, adapting to changing environmental demands requires a process of dynamical adjustments of such cognitive control in goal-directed behavior, which is referred to as “adaptive control”. It has been suggested that there are explicit distinctions between cognitive control processes and adaptive control processes. Specifically, cognitive control processes (e.g., conflict control) have been considered as more static, time-invariant processes, whereas adaptive control processes (e.g., adaptation to conflict control) reflect trial-to-trial conflict-triggered adjustments of cognitive control processes and research on adaptive control is primarily concerned with how cognitive control processes are regulated in a dynamic and time-varying manner (Braem et al., 2019).

Plenty of studies have shown that higher performance-contingent incentives can facilitate a set of high-order cognitive/adaptive control processes, including inhibitory control (Diao et al., 2016; Herrera et al., 2014; Zhang et al., 2016), task-switching (Etzel et al., 2015; Hall-McMaster et al., 2019; Wisniewski et al., 2015), conflict control (Krebs et al., 2010; Padmala & Pessoa, 2011), and adaptation to conflict control (Braem et al., 2012, 2014). While reward is generally associated with performance enhancement, the presence of conflicting information is widely known to impede human behavior, as illustrated by conflict tasks such as flanker, Stroop, and Simon tasks (Eriksen & Eriksen, 1974; Simon & Rudell, 1967; Stroop, 1935). In these conflict tasks, target stimulus and irrelevant stimulus are presented simultaneously, and participants are required to resolve the sort of conflict by virtue of cognitive control. For example, in an arrow flanker task, participants are instructed to react to the direction of a central target arrow that is flanked by arrows with directions that are either the same (i.e., congruent trial) or opposite (i.e., incongruent trial) to the central target arrow. Human performance on the target can be interfered by the flankers, leading to prolonged reaction times (RTs) and more errors in incongruent trials than in congruent trials, which is called the “congruency effect” or “conflict effect”. Crucially, the congruency of the previous trial can regulate human performance on subsequent trial in conflict tasks. For instance, the reaction time difference (i.e., conflict effect) between incongruent trial (iI trial) and congruent trial (iC trial) following incongruent trial was observed to be smaller than that between incongruent trial (cI trial) and congruent trial (cC trial) following congruent trial. The trial-to-trial adaptations of conflict control are commonly referred to as the “sequential congruency effect” or “conflict adaptation effect” (Gratton & Gabriele, 1992).

The conflict adaptation effect can be interpreted using several different approaches. The *conflict-monitoring account* assumes a top-down, cognitive control adaptation (Botvinick et al., 2001). In this view, conflict adaptation is derived from the increased recruitment of control (i.e., greater task-relevant focus) when previous conflict was detected, which is conducive to conflict resolution in the current trial. However, the *feature-integration* or *feature-priming account* argue a more basic, bottom-up process through which conflict adaptation can be caused by response/stimulus feature repetition/alternation (Hommel et al., 2004; Mayr et al., 2003). The account emphasizes that stimuli and response features are intergrated in the same episodic memory representation (i.e., feature binding), resulting in a more rapid response when features are identical or different between previous and current trials (e.g., cC and iI trials) compared to when features are partial repetition of previous and curent trial (e.g., iC and cI trials), leading to conflict adaptation. In contrast, the *reinforcement learning account* (also called *adaptation-by-binding account*) stresses that conflict adaptation results from the interaction of arousal signals induced by conflict processes and ongoing associative learning of specific stimulus-response associations (Verguts & Notebaert, 2008, 2009). According to this account, the conflict information of an incongruent trial can be captured by a conflict detection system, which triggers an arousal response in the locus coeruleus. The locus coeruleus can regulate the ongoing associative learning (Hebbian learning), and therefore, affect the binding of task-relevant representations. The enhanced Hebbian learning in incongruent trials leads to a better adaptation of cognitive control. Recently, Dignath et al. (2020) has proposed the *affective signaling account* in which conflict adaptation is triggered by negative affect rather than by conflict per se. According to this account, experiencing a conflict is always associated with negative affect and avoidance motivation, which can be alleviated by increased control in the following conflict trial, resulting in adaptation to conflict. Although the mechanisms underlying conflict adaptation are still debated, it is widely agreed that conflict-triggered adaptive control processes represent a kind of dynamic update of conflict control, and play a critical role in changing environment demands (Braem et al., 2019; Egner, 2007).

Conflict adaptation has been found to be promoted by motivational manipulations (e.g., reward feedback, performance-contingent and performance-noncontingent rewards) in most but not all studies (e.g., Braem et al., 2012, 2014; Chiew & Braver, 2014; Soutschek et al., 2014 for supporting evidence; but see van Steenbergen et al., 2009 for impaired conflict adaptation exerted by reward). Several lines of empirical literatures and reviews have dissected the differential findings in terms of reward-induced affection and motivation, and tried to establish unified theoretical frameworks to reconcile the discrepancy (Dreisbach & Fischer, 2012; Notebaert & Braem, 2015). It is noteworthy that earlier studies concerning the phasic influence of reward on conflict adaptation by setting high-value and low-value reward (or no-reward) signals are intermixed in the same blocks (Braem et al., 2012, 2014; Stürmer et al., 2011), which leads to the neglect of the tonic effect of reward on conflict adaptation. Additionally, it has been suggested that reward modulations of cognitive control may be influenced by inter-trial changes in the performance-contingent reward magnitude (Frober & Dreisbach, 2016; Shen & Chun, 2011). For instance, Shen and Chun (2011) developed a rewarded cued task-switching paradigm in which high- or low-value reward were presented randomly and observed increased cognitive flexibility for high-value reward following low-value reward than for that following high-value reward.

Thus, the observations of reward modulations of conflict adaptation in prior work may be contaminated by the influence of trial-to-trial changes in reward magnitude. In order to rule out the potential interference, a novel experimental design in which cognitive tasks were manipulated with reward presented at a block level may be a better candidate for detecting the effect of reward on adaptive control (e.g., conflict adaptation).

It is noteworthy that conventional perspectives hold that cognitive/adaptive control processes require consciousness (Dehaene & Naccache, 2001; Eimer & Schlaghecken, 2002). However, recent evidence has increasingly shown that both cognitive control and adaptive control processes can operate in the absence of conscious awareness in masked priming tasks. The supporting evidence includes empirical research results on several components of cognitive control processes (e.g., inhibitory control: Hughes et al., 2009; van Gaal et al., 2008, 2010b; task-switching: De Pisapia et al., 2012; Lau & Passingham, 2007; Reuss et al., 2011; and conflict control: Jiang et al., 2016; Wang et al., 2013) and adaptive control process (e.g., conflict adaptation: Desender et al., 2013; Jiang et al., 2015; van Gaal et al., 2010a). However, whether these unconsciously triggered cognitive control processes can be influenced by reward is largely unknown. In our recent study, we adopted a masked priming of go/no-go task manipulated with high- or low-value performance-contingent reward presented at the block level to investigate how reward affects unconsciously triggered cognitive control processes and observed that high-value rewards strengthened unconsciously triggered cognitive control processes (e.g, inhibitory control) compared to low-value rewards, as evidenced by a pronounced P3 component (Diao et al., 2016). Given that there are explicit distinctions between cognitive and adaptive control processes, whether unconsciously triggered adaptive control processes can be affected by reward is still an open question.

To address this question, we conducted two experiments. In Experiment 1, we used a rewarded masked flanker-like priming task to detect whether unconsciously triggered adaptive control processes can be affected by reward. In Experiment 2, we adopted a rewarded masked Stroop-like priming task to investigate whether this reward effect still exists when controlling the stimulus-response repetitions.

## Experiment 1

### Method

#### Participants

Thirty right-handed undergraduate students (16 females, age range = 18‒23 years, *M* = 20.12 years, *SD* = 1.18) were recruited to participate in the experiment. All participants were right-handed, had normal or corrected-to-normal vision, and had no history of physical or mental illness. One participant was excluded due to quitting halfway through the study. Both Experiments 1 and 2 were approved by the ethics committee of the Academy of Neuroeconomics and Neuromanagement at Ningbo University, and written informed consent document was obtained from each participant in compliance with the tenets of the Declaration of Helsinki.

#### Stimuli and Apparatus

In Experiment 1, we adopted a masked flanker-like priming task adapted from van Gaal et al. (2010a), in which the primes were white left-pointing or right-pointing arrows (visual angle: 0.74° × 0.25°) and the targets were somewhat larger white left- or right-pointing arrows (visual angle: 1.45° × 0.49°). All stimuli were presented at the center of a 16-inch View Sonic CRT monitor (frequency 60 Hz, resolution 1024 × 768, framerate about 16.7 ms) with E-prime software package (version 3.0; Psychology Software Tools, Inc., Pittsburgh, PA, USA). The participants were seated approximately 70 cm away from the computer screen.

#### Procedure

The main experiment comprised of 24 blocks of conscious tasks (the primes were weakly masked in the conscious task) in which half of the blocks were manipulated with high-value rewards and another half of blocks were manipulated with low-value rewards, and 24 blocks of unconscious tasks (the primes were strongly masked in the unconscious task) in which half of the blocks were manipulated with high-value rewards and another half of the blocks were manipulated with low-value rewards, with 36 trials in each block. A high- (10 points) or low-value (1 point) reward signal was presented for 3000 ms at the beginning of each block, indicating that one would earn either 10 points or 1 point for their correct and fast response on each trial in high- or low-value reward blocks, respectively. High- and low-value reward blocks were presented randomly.

In each trial in the unconscious task, a white left-pointing or right-pointing arrow (duration: 17 ms, serving as prime) was presented against a black background, followed by a blank screen (duration: 33 ms), a somewhat larger left- or right-pointing arrow (duration: 133 ms, serving as target), another blank screen (duration: 1000 ms), and a feedback screen (500 ms). The prime fitted exactly within the inner contour of the target, and therefore the target served as a meta-contrast mask, strongly reducing stimulus visibility (Breitmeyer et al., 1984). In the unconscious task, participants were not told about the presence of the prime, but were told that there was a flash preceding the target and that they should focus their attention on the target.

**Figure 1.**
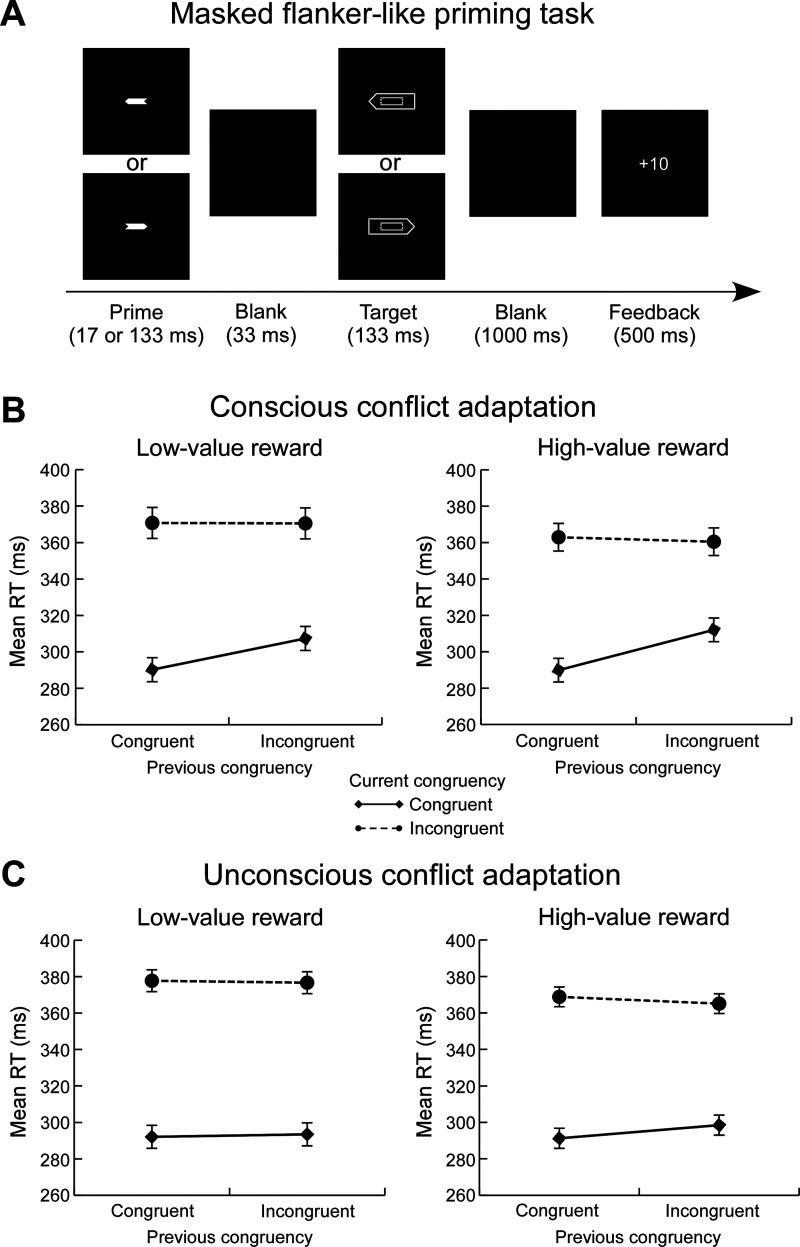
Experimental design of the masked flanker-like priming task in experiment 1 (A). The figures of experimental results illustrate how reward modulates consciously triggered conflict adaptation (B) and unconsciously triggered conflict adaptation (C). Error bars represent the standard error across participants. RT = reaction time.

In each trial in the conscious task, the stimuli and procedure were the same as in the unconscious task, with the exception that the duration of the prime was 133 ms to ensure the visibility of the prime. To avoid participants becoming aware of the presence of prime in the unconscious task, the unconscious task was always carried out first for each participant (Desender et al., 2013). Figure 1A shows the experimental design.

Participants were informed to focus their attention on the target and press the “*f*” key for the left-pointing target with the left index finger, and to press the “*j*” key for the right-pointing target with the right index finger on a standard QWER-keyboard. Participants received performance-contingent rewards when their responses were correct and fast enough (for details, see the section of *reward manipulation and feedback* shown below). In each block, half of the trials were congruent (i.e., the direction of the prime and target were the same) and the other half were incongruent (i.e., the direction of the prime and target were opposite), and all trials were presented randomly. The inter-trial interval had a variable duration (1000–1500 ms).

Upon completion of one practice block of 40 trials and 48 experimental blocks of 40 trials, participants were required to finish a forced-choice discrimination task to detect the visibility of the prime. The discrimination task consisted of one block of conscious tasks and one block of unconscious tasks with 72 trials in each block, which was identical to the experimental task. Participants were informed to ignore the target, but try their best to discriminate the direction of the prime. They were instructed to press the “*v*” key for the left-pointing prime, to press the “*n*” key for the right-pointing prime, and were asked to guess if they were not sure about the identity of the prime.

#### Reward Manipulation and Feedback

Participants were told to respond as quickly and accurately as possible within 1000 ms to receive a reward. We used an adaptive reaction time (RT) threshold to keep participants constantly motivated (Wisniewski et al., 2015). More precisely, we extracted the RTs from the last 25 trials with correct responses for an RT distribution, and regarded the 70th percentile of the RT distribution as the RT threshold for the current trial, and so on. This manipulation would lead to participants being too slow to earn a reward in approximately 30% of all trials, even if they had a correct response. For the RT thresholds of the first 25 trials in experimental task, we used the RTs of the last 25 trials with correct responses in the practice task to determine the RT thresholds. The RT distributions for high- and low-value reward conditions were calculated specifically to avoid potential cross-contamination. Participants received a warning feedback (duration: 500 ms) at the end of each trial when their response was too slow (a yellow cross as feedback), wrong (a red cross as feedback), or a miss (a blue cross as feedback) and received “ + 10 points” or “ + 1 point” as feedback when their response was correct and fast enough. They were informed that the points accumulated during the experiment would be proportionally exchanged into money (200 points exchanged for 1 Chinese Yuan, about $0.14), and they would achieve the money they earned after the experiment.

### Results

#### Prime Discrimination

All participants reported that they could not discriminate the strongly masked primes, and the mean accuracy of the strongly masked prime was 50.6% (*SD* = 0.03). Moreover, we calculated the discrimination performance (*d′*) of strongly masked primes using the formula [*Z*_*hit rate*_ −*Z _false alarm rate_*] and found that *d′* did not differ significantly from zero (*d′* = .02, *t*_(28)_ = .93, *p* = .36), suggesting that there is no evidence supporting the conscious discrimination of strongly masked primes in the force-choice discrimination task, implying that strongly masked primes in Experiment 1 were presented in an unconscious manner. In contrast, the mean accuracy of the weakly masked prime was 90.5% (*SD* = 0.02), and the discrimination performance of weakly masked primes differed significantly from zero (*d′* = 1.76, *t*_(28)_ = 10.81, *p *< .001), suggesting that weakly masked primes in Experiment 1 were presented in a conscious manner. Therefore, we will refer to the weakly vs. strongly masked prime tasks as conscious task vs. unconscious tasks from now on.

#### Reaction Times (RTs)

The mean RTs (trials with incorrect responses or preceded by an incorrect response, the first trial of each block, and trials with RTs beyond three *SD*s were detected under each treatment, and a total of 7.39% of trials were excluded) were included in the analysis. Then, a repeated-measures analysis of variance (rm-ANOVA) was conducted with 2 (reward: high versus low value) × 2 (task: conscious task vs. unconscious task) × 2 (previous congruency: congruent vs. incongruent) × 2 (current congruency: congruent vs. incongruent) as within-subject factors.

Table 1 shows the mean RTs as a function of reward, task, previous congruency, and current congruency in Experiment 1. There was a significant congruency effect (*F*_(1, 28)_ = 445.30, *p *< .001, $\eta_{p}^{2}$ = .94), which interacted with previous congruency (*F*_(1, 28)_ = 88.05, *p *< .001, $\eta_{p}^{2}$ = .76), indicating an *overall conflict adaptation effect*. We also observed a significant interaction between task, previous congruency, and current congruency (*F*_(1, 28)_ = 24.69, *p *< .001, $\eta_{p}^{2}$ = .47), indicating a stronger conflict adaptation in the conscious task compared to that in the unconscious task (19.51 ± 13.63 ms *vs.* 5.21 ± 8.80 ms). Importantly, the interaction between reward, previous congruency, and current congruency was significant (*F*_(1, 28)_ = 14.53, *p* = .001, $\eta_{p}^{2}$ = .34), demonstrating that reward may modulate adaptation to conflict. However, the four-way interaction effect between reward, task, previous congruency, and current congruency was not significant (*F *< 1). To further detect how reward influenced the conscious and unconscious conflict adaptation effect, we conducted a planned rm-ANOVA with reward, previous congruency, and current congruency as within-subject factors for conscious and unconscious tasks, respectively.

**Table 1.** Mean RTs (in *ms*) and error rates (in *%*) for each condition in experiment 1 (*M* ± *SD*).

Task	Reward	cC	cI	iC	iI	Conflict adaptation
RTs (*ms*)						
Conscious task	Low-value	290.23 ± 36.04	370.76 ± 45.93	307.36 ± 35.78	370.51 ± 45.98	17.37 ± 14.63
	High-value	289.88 ± 34.34	362.97 ± 43.49	312.04 ± 35.47	360.49 ± 41.41	24.64 ± 14.85
Unconscious task	Low-value	292.09 ± 31.43	377.75 ± 36.72	293.45 ± 29.48	376.69 ± 35.46	2.42 ± 11.94
	High-value	291.27 ± 27.09	368.81 ± 32.80	298.50 ± 24.90	365.08 ± 34.33	10.96 ± 16.81
Error rates (%)						
Conscious task	Low-value	3.24 ± 4.05	16.55 ± 12.8	4.69 ± 5.94	4.52 ± 11.72	13.48 ± 8.45
	High-value	3.24 ± 3.75	16.79 ± 9.96	4.38 ± 5.81	5.62 ± 11.55	12.31 ± 8.89
Unconscious task	Low-value	2.41 ± 4.33	7.90 ± 13.74	2.38 ± 4.23	5.14 ± 13.30	2.72 ± 6.46
	High-value	1.72 ± 3.11	10.14 ± 11.79	2.24 ± 4.66	7.03 ± 13.26	3.62 ± 5.90

In the conscious task, there was a significant congruency effect (*F*_(1, 28)_ = 33.60, *p *< .001, $\eta_{p}^{2}$ = .55), which interacted with previous congruency (*F*_(1, 28)_ = 68.88, *p *< .001, $\eta_{p}^{2}$ = .71), indicating a significant *conscious conflict adaptation effect*. Importantly, there was a significant three-way interaction between reward, previous congruency, and current congruency (*F*_(1, 28)_ = 12.15, *p* = .002, $\eta_{p}^{2}$ = .30), suggesting that the conscious conflict adaptation can be modulated by reward. Follow-up tests showed that there were significant differences between cC trials and iC trials in both high- and low-value reward conditions (high-value reward condition: *F*_(1, 28)_ = 79.55, *p *< .001, $\eta_{p}^{2}$ = .74; low-value reward condition: *F*_(1, 28)_ = 61.56, *p *< .001, $\eta_{p}^{2}$ = .69). No other significant effect was observed (*p*s > .05). Next, we calculated the conscious conflict adaptation effect in the high- and low-value reward conditions using the formula [(cI − cC) − (iI − iC)], and observed that participants elicited a larger conscious conflict adaptation for the high-value reward than for the low-value reward (24.64 ± 14.85 ms *vs.* 17.37 ± 14.63 ms; *F*_(1, 28)_ = 12.15, *p* = .002, $\eta_{p}^{2}$ = .30), indicating the reward-induced enhancement of consciously triggered adaptive control processes (see Figure 1B).

In the unconscious task, there was a significant congruency effect (*F*_(1, 28)_ = 841.00, *p *< .001, $\eta_{p}^{2}$ = .97), which interacted with previous congruency (*F*_(1, 28)_ = 13.71, *p* = .001, $\eta_{p}^{2}$ = .33), indicating a significant *unconscious conflict adaptation effect*. Importantly, there was a significant three-way interaction between reward, previous congruency, and current congruency (*F*_(1, 28)_ = 4.49, *p* = .043, $\eta_{p}^{2}$ = .14), suggesting that unconscious conflict adaptation can be modulated by reward. Follow-up tests showed that there was a significant difference between cC trials and iC trials in the high-value reward condition (*F*_(1, 28)_ = 26.00, *p *< .001, $\eta_{p}^{2}$ = .48). No other significant effect was observed (*p*s > .10). Next, we calculated the unconscious conflict adaptation effect in the high- and low-value reward conditions using the formula [(cI − cC) − (iI − iC)], and observed that participants also elicited a larger unconscious conflict adaptation for high-value reward than for low-value reward (10.96 ± 16.81 ms *vs.* 2.42 ± 11.94 ms; *F*_(1, 28)_ = 4.49, *p* = .043, $\eta_{p}^{2}$ = .14), indicating the reward-induced increment of unconsciously triggered adaptive control processes (see Figure 1C).

#### Error Rates

Table 1 also shows the mean RTs as a function of reward, task, previous congruency, and current congruency in Experiment 1. An rm-ANOVA was carried out with reward, task, previous congruency, and current congruency as within-subject factors. The results showed a significant congruency effect (*F*_(1, 28)_ = 461.10, *p *< .001, $\eta_{p}^{2}$ = .94), which interacted with previous congruency (*F*_(1, 28)_ = 108.60, *p *< .001, $\eta_{p}^{2}$ = .79), indicating an *overall conflict adaptation effect*. However, we did not find a four-way interaction between reward, task, previous congruency, and current congruency (*F *< 1). Next, we conducted an rm-ANOVA with reward, previous congruency, and current congruency as within-subject factors for the conscious and unconscious tasks, respectively, and the results also did not show any interactions (*F*s < 1).

### Discussion

In Experiment 1, we developed a rewarded masked flanker-like task in which high- or low-value contingent performance rewards were presented at the block level to detect the influence of reward on unconsciously triggered conflict adaptation. The results showed that both consciously and unconsciously triggered conflict adaptation were increased by high-value rewards. However, it is noteworthy that conflict adaptation may be caused by low-level perceptual processing (e.g., response/stimulus feature repetitions) or contingency effects rather than higher-order adaptive/cognitive control (Duthoo et al., 2014; Hommel et al., 2004; Mayr et al., 2003), leading to an artefact of conflict adaptation in conflict tasks (Egner, 2007). Previously, van Gaal et al. (2010a) adopted a similar masked flanker-like priming task and observed that the conflict adaptation still exists when partially controlling the stimulus-response repetitions, which is still not enough to be convincing as the masked flanker-like priming task in both van Gaal's and our study only contained two stimulus-response mappings and the repetition effect in conflict adaptation cannot be completely controlled (Puccioni & Vallesi, 2012; Zeng et al., 2017).

These ambiguous findings indicate that conclusions about the effect of reward on consciously and unconsciously triggered conflict adaptation should be cautiously drawn unless the potential contamination of stimulus-response repetitions was completely excluded. Therefore, we developed a rewarded masked Stroop-like priming task in Experiment 2 to detect whether the reward-induced enhancement of consciously and unconsciously triggered conflict adaptation still exists when controlling stimulus-response repetitions.

## Experiment 2

### Method

#### Participants

Thirty-four right-handed undergraduate students (20 females, age range = 18‒23 years, *M* = 19.66 years, *SD* = 1.17) were recruited to participate in the experiment. All participants were right-handed, had normal or corrected-to-normal vision, and had no history of physical or mental illness. One participant was excluded due to quitting halfway through the study.

#### Stimuli and Apparatus

**Figure 2.**
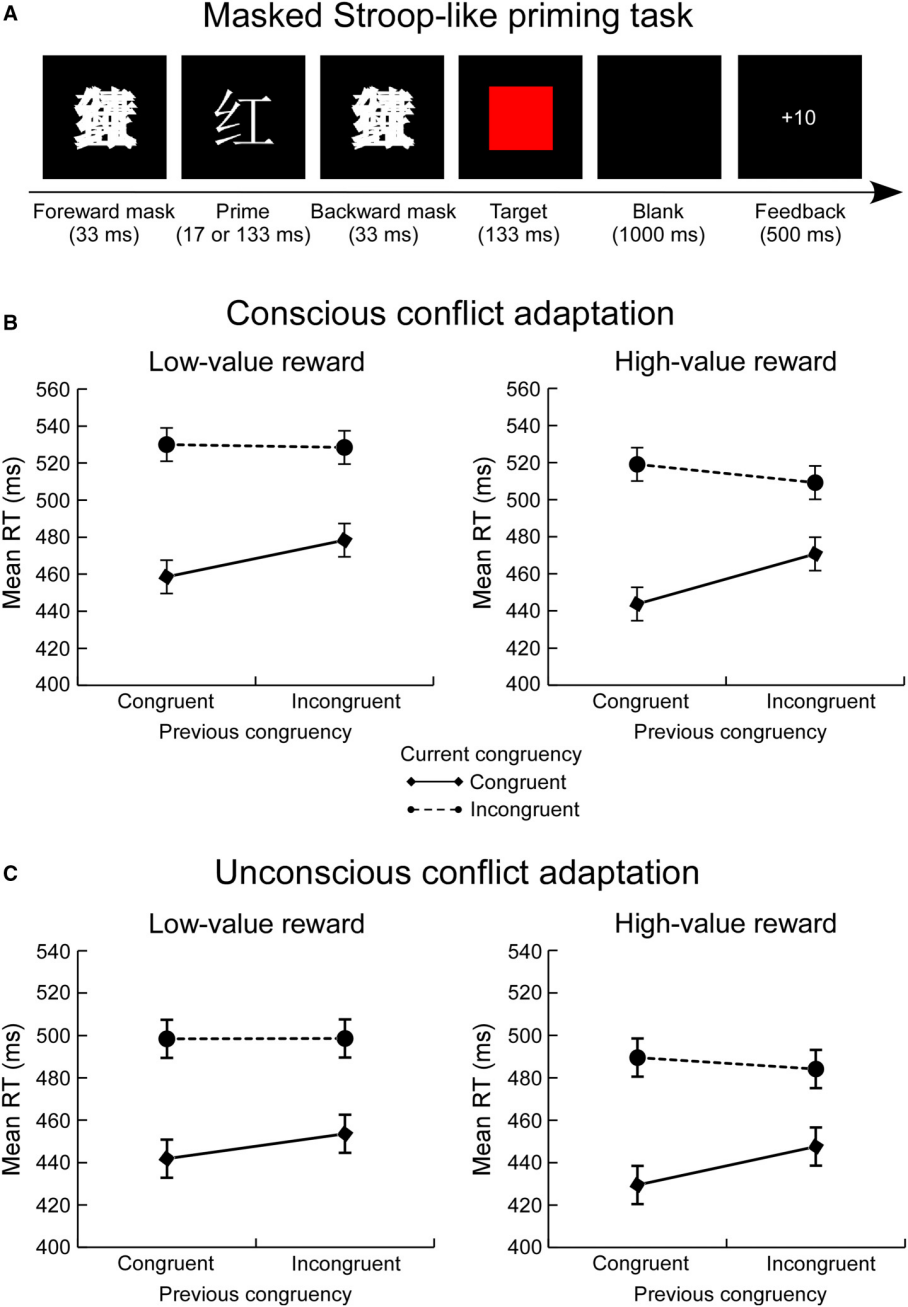
Experimental design of the masked Stroop-like priming task in experiment 2 (A). The figures of experimental results illustrate how reward modulates consciously triggered conflict adaptation (B) and unconsciously triggered conflict adaptation (C). Error bars represent the standard error across participants. RT = reaction time.

In Experiment 2, we adopted a masked Stroop-like priming task adapted from Jiang et al. (2016) in which the primes were four white Chinese color words (红, 黄, 蓝, and 绿, which correspond to red, yellow, blue, and green, appearing in Song font in 36-point size and extending a visual angle of 1.05° × 1.05°). The masks were made by overlapping four color words and enlarging the image to 1.1 × as large as the prime (visual angle: 1.16° × 1.16°). The targets were four patches colored red, yellow, blue, or green, which was the same size as the masks (Figure 2A). All stimuli were presented at the center of a 16-inch View Sonic CRT monitor (frequency 60 Hz, resolution 1024 × 768, framerate about 16.7 ms) with E-prime 3.0 software (version 3.0; Psychology Software Tools, Inc., Pittsburgh, PA, USA). The participants were seated approximately 70 cm away from the computer screen.

#### Procedure

The experimental procedure of Experiment 2 was identical to that of Experiment 1. High- and low-value reward blocks were randomly presented in Experiment 2. Figure 2A shows the experimental design.

Participants were instructed to focus their attention on the target and to press the “*d*” key for the red patch with the left middle finger, to press the “*f*” key for the yellow patch with the left index finger, to press the “*j*” key for the blue patch with the right index finger, and to press the “*k*” key for the green patch with the right middle finger on a standard QWER-keyboard. They were trained for 10 min to remember the color-key mappings, and all of them achieved ≥95% accuracy during the training. Participants received performance-contingent rewards when their responses were correct and fast enough. In each block, half of the trials were congruent (i.e., the Chinese color word matched the color of the target) and the other half were incongruent (i.e., the Chinese color word did not match the color of the target). Critically, to prevent potential stimuli-response repetitions and contingency learning effects, we carried out two prime-target combinations, yellow/blue and red/green. The two prime-target combinations were presented in alternating order, so that no current-trial color or response was repeated in the subsequent trial (Landman & van Steenbergen, 2020). The inter-trial interval had a variable duration (1000–1500 ms).

Upon completion of one practice block of 40 trials and 48 experimental blocks of 40 trials, participants were required to finish a forced-choice discrimination task to detect the visibility of the prime. The discrimination task consisted of one block of conscious tasks and one block of unconscious tasks with 72 trials in each block, which was identical to the experimental task. Participants were informed to ignore the target but try their best to discriminate Chinese color words. They were informed to press the “*c*” key for “red”, the “*v*” key for “yellow”, the “*n*” key for “blue”, the “*m*” key for “green”, and to guess if they were not sure about the identity of the prime.

#### Reward Manipulation and Feedback

The reward manipulation and feedback were identical to those in Experiment 1.

### Results

#### Prime Discrimination

All participants reported that they could not discriminate strongly masked Chinese color words. The mean accuracy of strongly masked prime discrimination was 25.1% (*SD* = 0.03), and the discrimination performance of the strongly masked prime (*d′*) did not differ significantly from zero (*d′* = .03, *t*_(32)_ = .41, *p *> .68), suggesting that there is no evidence supporting the conscious discrimination of strongly masked primes in the force-choice discrimination task, implying that strongly masked primes in Experiment 2 were presented in an unconscious manner. In contrast, the mean accuracy for the weakly masked prime discrimination was 88.4% (*SD* = 0.02), and the discrimination performance differed significantly from zero (*d′* = 1.68, *t*_(32)_ = 10.12, *p *< .001), suggesting that the weakly masked primes in Experiment 2 were presented consciously. Therefore, we will refer to the two task conditions as conscious task vs. unconscious task from now on.

#### Reaction Times (RTs)

The mean RTs (trials with incorrect responses or preceded by an incorrect response, the first trial of each block, and trials with RTs beyond three *SD*s were detected under each treatment, and a total of 9.68% of trials were excluded) were included in the analysis. Then, an rm-ANOVA was performed with 2 (*reward*: high *vs.* low value) × 2 (*task*: conscious task *vs.* unconscious task) × 2 (*previous congruency*: congruent *vs.* incongruent) × 2 (*current congruency*: congruent *vs.* incongruent) as within-subject factors.

Table 2 shows the mean RTs as a function of task, reward, previous congruency, and current congruency in Experiment 2. Results showed that reward had a significant main effect (*F*_(1, 32)_ = 13.20, *p* = .001, $\eta_{p}^{2}$ = .29), suggesting that participants elicited a faster response for high-value rewards than for low-value rewards (474.17 ± 48.68 ms *vs.* 486.07 ± 52.59 ms). There was a significant congruency effect (*F*_(1, 32)_ = 975.61, *p *< .001, $\eta_{p}^{2}$ = .96), which interacted with previous congruency (*F*_(1, 32)_ = 74.97, *p *< .001, $\eta_{p}^{2}$ = .70), indicating an *overall conflict adaptation effect*. We observed a marginally significant interaction between task, previous congruency, and current congruency (*F*_(1, 28)_ = 3.88, *p* = .058, $\eta_{p}^{2}$ = .11), indicating a stronger conflict adaptation in the conscious task compared to that in the unconscious task (29.43 ± 17.41 ms *vs.* 17.54 ± 20.99 ms). Additionally, the interaction effect between reward, previous congruency, and current congruency was significant (*F*_(1, 32)_ = 11.67, *p* = .002, $\eta_{p}^{2}$ = .27), indicating that reward modulates adaptation to conflict. However, the four-way interaction effect between task, reward, previous congruency, and current congruency was not significant (*F *< 1). We carried out a planned rm-ANOVA with reward, previous congruency, and current congruency as within-subject factors for conscious and unconscious tasks, respectively.

**Table 2.** Mean RTs (in *ms*) and error rates (in *%*) for each condition in experiment 2 (*M* ± *SD*).

Task	Reward	cC	cI	iC	iI	Conflict adaptation
RTs (*ms*)						
Conscious task	Low-value	458.57 ± 57.95	530.68 ± 54.73	478.38 ± 58.90	528.47 ± 54.31	22.01 ± 21.43
	High-value	443.76 ± 64.47	519.06 ± 60.16	470.76 ± 65.57	509.20 ± 60.33	36.85 ± 24.88
Unconscious task	Low-value	441.83 ± 65.80	498.42 ± 63.93	453.59 ± 70.34	498.60 ± 60.65	11.59 ± 33.34
	High-value	429.44 ± 51.80	489.49 ± 57.21	447.56 ± 54.55	484.10 ± 49.85	23.51 ± 31.31
Error rates (*%*)						
Conscious task	Low-value	4.72 ± 5.72	9.75 ± 11.53	5.83 ± 6.31	6.19 ± 9.43	4.67 ± 9.92
	High-value	4.83 ± 5.53	9.67 ± 11.67	7.22 ± 6.64	7.67 ± 8.82	4.39 ± 10.68
Unconscious task	Low-value	7.89 ± 6.54	13.03 ± 12.20	11.44 ± 7.82	12.03 ± 11.14	4.56 ± 11.61
	High-value	7.58 ± 7.50	14.03 ± 12.10	10.61 ± 6.57	12.28 ± 11.28	4.78 ± 9.11

In the conscious task, there was a significant congruency effect (*F*_(1, 32)_ = 458.40, *p *< .001, $\eta_{p}^{2}$ = .94), which interacted with previous congruency (*F*_(1, 32)_ = 94.32, *p *< .001, $\eta_{p}^{2}$ = .75), indicating an *overall conscious conflict adaptation effect*. Critically, we observed a significant three-way interaction between reward, previous congruency, and current congruency (*F*_(1, 32)_ = 7.70, *p *< .01, $\eta_{p}^{2}$ = .19), indicating that the conscious conflict adaptation effect can be affected by reward. Follow-up tests showed that there were significant differences between cC trials and iC trials in both high- and low-value reward conditions (high-value reward condition: *F*_(1, 32)_ = 58.15, *p *< .001, $\eta_{p}^{2}$ = .65; low-value reward condition: *F*_(1, 32)_ = 31.90, *p *< .001, $\eta_{p}^{2}$ = .49). No other significant effect was observed (*p*s > .05). Next, we calculated the conscious conflict adaptation effect in high-value reward condition and low-value reward condition and observed that participants elicited a larger conscious conflict adaptation for high-value rewards than for low-value rewards (36.85 ± 24.87 ms *vs.* 22.02 ± 21.43 ms; *F*_(1, 32)_ = 7.70, *p *< .01, $\eta_{p}^{2}$ = .19), confirming the reward-induced enhancement of consciously triggered adaptive control processes (see Figure 2B).

In the unconscious task, there was a significant congruency effect (*F*_(1, 32)_ = 221.50, *p *< .001, $\eta_{p}^{2}$ = .87), which interacted with previous congruency (*F*_(1, 32)_ = 12.96, *p* = .001, $\eta_{p}^{2}$ = .29), suggesting an overall *unconscious conflict adaptation effect*. Critically, we observed a significant three-way interaction between reward, previous congruency, and current congruency (*F*_(1, 32)_ = 4.48, *p* = .042, $\eta_{p}^{2}$ = .12), suggesting that the unconscious conflict adaptation effect can be modulated by reward. Follow-up tests showed that there were significant differences between cC trials and iC trials in both high- and low-value reward conditions (high-value reward condition: *F*_(1, 32)_ = 30.10, *p *< .001, $\eta_{p}^{2}$ = .49; low-value reward condition: *F*_(1, 32)_ = 8.77, *p* = .006, $\eta_{p}^{2}$ = .22). No other significant effect was observed (*p*s > .13). Next, we calculated unconscious conflict adaptation in the high- and low-value reward conditions and observed that participants elicited a larger unconscious conflict adaptation for high-value rewards than for low-value rewards (23.51 ± 31.31 ms *vs.* 11.59 ± 33.34 ms; *F*_(1, 32)_ = 4.48, *p* = .042, $\eta_{p}^{2}$ = .12), indicating the reward-induced improvement of unconsciously triggered adaptive control processes (see Figure 2C).

#### Error Rates

Table 2 also shows the error rates as a function of task, reward, previous congruency, and current congruency in Experiment 2. An rm-ANOVA was conducted with task, reward, previous congruency, and current congruency as within-subject factors. The results showed a significant congruency effect (*F*_(1, 32)_ = 212.10, *p *< .001, $\eta_{p}^{2}$ = .86), which interacted with previous congruency (*F*_(1, 32)_ = 65.61, *p *< .001, $\eta_{p}^{2}$ = .65), indicating an *overall conflict adaptation effect*. We did not find a four-way interaction between reward, task, previous congruency, and current congruency (*F *< 1). Next, we conducted an rm-ANOVA with reward, previous congruency, and current congruency as within-subject factors for conscious and unconscious tasks, respectively, and the results also did not show any interactions (*F*s < 1).

### Discussion

In Experiment 2, we developed a rewarded masked Stroop-like task in which high- or low-value contingent performance rewards were presented at the block level to detect the effect of reward on unconscious adaptation to conflict, excluding the potential contamination of stimulus-response repetitions. The findings of Experiment 2 replicated the results of Experiment 1 and showed that high-value rewards can enhance both consciously and unconsciously triggered conflict adaptation when controlling the stimulus-response repetitions, demonstrating that the (un)consciously triggered conflict adaptation effect was mainly driven by higher-order adaptive control processes (Jiang et al., 2015; van Gaal et al., 2010a). More importantly, the observation of reward-induced enhancement of consciously triggered adaptive control to conflict is in line with previous work (Braem et al., 2012; Stürmer et al., 2011) and suggests that reward cannot only strengthen the more static cognitive control processes, but also facilitate the dynamic trial-to-trial adaptation of the conflict control process. Moreover, the reward-induced improvement of unconsciously triggered adaptive control processes demonstrates that the influence of external rewards on adaptive control processes is independent of conflict awareness.

## General Discussion

In the present study, we conducted two experiments to explore the effect of reward on consciously and unconsciously triggered adaptive control processes. In order to exclude the potential interruptions of reward manipulation on outcomes, we developed a mixed-reward manipulation method in the present study. Rewards were presented at the block level to eliminate the interruptions by the trial-by-trial changes in reward magnitude on human performance (Frober & Dreisbach, 2016; Shen & Chun, 2011). Additionally, in each block, we adopted a RT threshold for each rewarded trial to rule out practice effects and maintain individuals’ motivation (Wisniewski et al., 2015). In Experiment 1, participants performed a rewarded masked flanker-like priming task and showed stronger consciously and unconsciously triggered conflict adaptation for high-value rewards compared to low-value rewards. In Experiment 2, participants completed a rewarded masked Stroop-like priming task to eliminate potential contamination of stimulus-response repetitions. The reward-induced enhancements of adaptive control processes still exist when controlling the influence of stimulus-response repetitions. These findings showed that reward modulates adaptive control to conflict (i.e., conflict adaptation), irrespective of whether previous conflict can be experienced consciously. To our knowledge, this is the first study to reveal the effect of reward on adaptive control processes at different levels of conflict awareness.

Compared to prior work adopting reward and no-reward signals in conflict tasks and suggesting that reward (vs. no-reward) increases conscious conflict adaptation (Braem et al., 2012; Stürmer et al., 2011), our study went further to use high-value vs. low-value reward signals to compare the influence of different reward magnitudes on human higher-order adaptive control processes in the same motivational dimension. This kind of reward manipulation provides an effective method to investigate the tonic effect of reward on (un)conscious cognitive/adaptive control processes, which would enrich the approaches to explore the interactions between reward and cognitive/adaptive control processes (Yee & Braver, 2018).

The observations of facilitated human performance (as reflected by faster reaction times) in high-value reward blocks are in line with prior work holding that individuals exposed to higher rewards tend to persistently maintain a higher level of motivation, indicating the long-lasting effect of reward on human performance (Capa et al., 2013). Moreover, we observed that there were significant differences between cC trials and iC trials (cC trials have faster responses) but not between iI trials and cI trials in both two experiments, regardless of task condition (conscious task or unconscious task). This distinct pattern of differences in RT was consistent with previous studies in which authors observed non-significant differences between cI and iI trials, even though there was a conflict adaptation effect as defined by a smaller difference in RT between cI and cC trials compared to iI and iC trials (Freitas et al., 2007; Gratton & Gabriele, 1992; Ullsperger et al., 2005). These interesting findings may be due to the variation of attention through which individual's response speed will be slowed after incongruent trials and facilitated after congruent trials. Thus, we propose that both the speeding up effect for cI trials and the slowing effect for iI trials contribute to the non-significant differences between cI and iI trails (Lamers & Roelofs, 2011).

Our findings showed that participants exerted stronger conscious conflict adaptation in the high-value reward condition, suggesting that reward modulates adaptive control processes. These findings can be appropriately interpreted by the conflict-monitoring account (Botvinick et al., 2001). According to this account, conflict adaptation derived from the increased recruitment of control when previous conflict was detected, which is helpful to conflict resolution in the current trial. It seems likely that the prospect of higher reward is related to greater recruitment of attentional control processes and facilitate the processing of task-relevant stimulus information in conflict tasks (Krebs et al., 2010; Padmala & Pessoa, 2011). Therefore, participants tend to maintain motivation to repeat the status of enhanced attentional control process after experiencing a conflict trial, which facilitates responding in the subsequent conflict trial, resulting in an increased conflict adaptation effect.

Alternatively, our findings can also be interpreted using the reinforcement learning account (Verguts & Notebaert, 2008, 2009). According to this account, conflict-induced arousal response strengthens ongoing associative learning, and therefore, promotes task-relevant representations, which is conducive to adaptation to conflict. Combining the theoretical framework and our findings, we reasoned that reward may facilitate adaptations to conflict by enhancing ongoing associative learning and task-relevant representations. Moreover, our finding that participants elicited faster responses on the cC trials than on the iC trials are in line with the study of Braem et al. (2012), in which reward feedback was manipulated to detect the transient effect of reward on trial-to-trial adaptations to conflict and the authors found that the participants elicited faster responses on the cC trials than on the iC trials in the reward context, implying that the task-relevant associations can be strengthened by reward signals.

It is noteworthy that our findings provide nonsupport for the affective signaling account (Dignath et al., 2020). The affective signaling account suggests that conflict adaptation effect is caused by the negative affect rather than the conflict per se. According to the account, external reward manipulation (which is often assumed to be associated with positive affect) is more likely to weaken/counteract the negative affect, leading to a decrease in conflict adaptation, which is opposite to our findings of reward-induced increment of conflict adaptation. The distinction may be due to the differential experimental design between the Dignath et al. (2020) and this study. In contrast to the Dignath et al. (2020) consisted of both reward and penalty trials manipulated by assigning reward and penalty to targets or distractors, there are only rewarded trials in our study (slower and incorrect responses/trials are not rewarded in the study and are further removed in the data analysis). Thus, the former aims to detect whether the motivation of the stimuli per se (always associated with negative feelings) can influence the conflict adaptation, whereas the later concerns whether the conflict adaptation can be affected by external rewards. Clearly, more research is needed to unravel the mechanisms underlying the two kinds of motivation-control interaction.

Crucially, we observed an enhanced unconsciously triggered conflict adaptation in the high-value reward condition, indicating reward-induced enhancements of unconscious adaptive control processes. These findings are consistent with our prior work in which an unconscious version of the Go/No-Go task was manipulated with high- or low-value reward presented at the block level, and the results showed that high-value reward facilitated consciously and unconsciously triggered inhibitory control processes, as reflected by the greater frontal P3 component (Diao et al., 2016). Combining the study of Diao et al. (2016) and our findings, we propose that reward, at the behavior level, not only affects consciously triggered cognitive and adaptive control processes, but also influences these processes operating in the absence of conscious awareness, although the consciously triggered cognitive and adaptive control processes were more pronounced. Our findings are also in accordance with the prior proposition that there is an interactive neural mechanism between reward (motivation) and unconscious cognitive and adaptive control networks through which reward information may permeate and activate a set of interconnected subcortical areas (e.g., inferior frontal cortex and medial frontal cortex) that have been implicated in unconsciously triggered cognitive (van Gaal et al., 2008, 2010b) and adaptive control processes (Jiang et al., 2015, 2016), which might facilitate the processing of unconsciously presented task-relevant stimuli. Compared to previous studies that mainly focused on the interactions between reward and consciously triggered cognitive and adaptive control processes, our research explored the impact of reward on unconsciously triggered trial-to-trial adaptation to conflict and demonstrated that the prospect of monetary rewards affects adaptive control processes, irrespective of conflict awareness. This is consistent with prior work suggesting that neural activity associated with consciously as well as unconsciously presented task-relevant stimuli could be influenced by external rewards (Pascucci et al., 2015; Pessiglione et al., 2008).

In conclusion, the purpose of this study was to investigate whether reward modulates unconsciously triggered adaptive control to conflict. We combined a masked flanker-like task (in Experiment 1) and a masked Stroop-like priming task (in Experiment 2) with high- and low-value rewards presented at the block level and observed that higher rewards enhanced both consciously and unconsciously triggered conflict adaptation, suggesting that reward modulates adaptive control processes to conflict, regardless of whether previous conflict can be experienced consciously. These findings expand our understanding of the relationship between reward (motivation), consciousness, and adaptive control processes.
